# Traditional and Virtual Cardiac Rehabilitation: Understanding the Changing Landscape of Cardiac Rehabilitation and the Implications on Patient Outcomes

**DOI:** 10.31083/RCM42725

**Published:** 2025-10-29

**Authors:** Sri Nuvvula, Adrian C. Chen, Amgad N. Makaryus

**Affiliations:** ^1^Departments of Medicine and Cardiology, Donald and Barbara Zucker School of Medicine at Hofstra/Northwell, Hempstead, NY 11549, USA; ^2^Department of Cardiology, Cardiovascular Institute at Northwell Health, Long Island, NY 11040, USA

**Keywords:** cardiac rehabilitation, virtual cardiac rehabilitation, quality of life, health disparities, patient preferences

## Abstract

Cardiac rehabilitation (CR) has been categorized as a class Ia recommendation for secondary prevention after major cardiac interventions or in patients with certain cardiac comorbidities. The benefits of CR have been established and range from reducing readmissions to improving quality of life. Given the increasing amount of literature on CR over the past few years and the evolution of this field, there is a need to synthesize these data. Thus, this review aims to combine the latest research findings to provide a comprehensive review of CR literature. We discuss the components needed to create a successful CR program, including individualized training plans, routine clinical assessments, exercise supervision, and nutritional assessments. Overall rates of CR utilization remain low. Therefore, we explore potential reasons for this underutilization observed in the literature, including CR deserts, under-referral, and the lack of education on benefits, time, and transportation. Moreover, we discuss solutions for underutilization that have been analyzed in the literature, including motivational interviewing, gender-specific regimens, transportation assistance, and automatic referrals. Realizing the underutilization of CR, we also assess virtual CR (VCR) and variations in various regimens within the programs. We compare exercise and body metrics, patient outcomes, feasibility, and patient preferences between VCR and traditional CR published in the literature. VCR does not appear to be inferior to conventional CR in many metrics, although more research is needed to compare the two modalities. We recommend that providers explain the outcomes of the two modalities and allow patients to choose the regimen that works best for them. We discuss how VCR may be better suited to patient populations with specific barriers to care. We also discuss the ongoing current CR trials, many of which are focused on solutions to underutilization. Lastly, we further discuss the remaining gaps in the CR literature and areas where future research could be beneficial, such as establishing large-scale VCR studies and studies focused on expanding CR indications.

## 1. Introduction 

Cardiac rehabilitation (CR) has become central to the field of cardiology with ever-expanding indications for patients suffering from cardiovascular disease. CR emerged in the mid-20th century after early studies challenged the longstanding dogma of prolonged bed rest following myocardial infarction (MI). Accumulating observational data and clinical trials from the 1970s through the 1990s showed that CR participation significantly reduced all-cause and cardiovascular mortality in patients who had an MI [1, 2]. Deriving from these landmark studies, the indications behind CR expanded to incorporate additional major adverse cardiovascular events and interventions.

Over the last few years, there has been increased interest in CR, and more literature has been published, especially in more nascent fields within CR like virtual CR (VCR). CR is known to have a large impact on patient outcomes and as a result, the American Heart Association and American College of Cardiology have established CR referral for secondary prevention after coronary artery bypass graft (CABG) surgery, MI, stable angina, percutaneous coronary intervention (PCI), symptomatic peripheral arterial disease (PAD), cardiac transplantation, chronic heart failure with reduced ejection fraction (HFrEF), and heart valve surgery [3]. CR has been shown to reduce readmissions and mortality [4]. CR has also been shown to decrease MI and improve quality of life metrics [5].

Given the increasing amount of CR literature over the past few years and the evolution of the field, there is a need to synthesize the literature in an understandable manner, especially for those who are not already familiar with CR. This review aims to combine the latest literature to provide a comprehensive review of CR. Education about CR is especially important given the large underutilization of CR within the field with only 16.3% of qualifying Medicare patients and 10.3% of Veterans Affairs patients participating [6]. We will discuss indications, changes in metrics, and improved patient outcomes of CR. We also aim to describe CR regimens and variations within the regimens. We also discuss the reasons for its underutilization and potential solutions for CR underutilization. Studies show similar outcomes of VCR to traditional CR, and we discuss the literature regarding VCR in detail [7, 8] and discuss variations within VCR regimens. We also compare exercise and body metrics, patient outcomes, feasibility, and patient preferences between VCR and traditional CR. We discuss how VCR may be better suited to patient populations with specific barriers to care. We also discuss current ongoing studies and discuss areas for future research within CR.

## 2. Literature Review

### 2.1 CR Methodology 

CR is a multi-component program designed for enhancing recovery after major adverse cardiovascular events and or intervention. Currently, the American Heart Association and American College of Cardiology endorse CR across a broad spectrum of indications, including acute coronary syndrome and stable angina, PAD, symptomatic HFrEF, PCI, post-CABG surgery, cardiac transplantation, and after valvular repair or replacement [3, 9, 10, 11]. Cardiovascular risk factor-based referrals for CR are also made, given the large amount of evidence regarding the impact of CR on these risk factors [12]. Specifically, the benefits of CR on patients with conditions, including diabetes mellitus, pulmonary disease, and metabolic syndrome have been described [12].

**Fig. 1.**
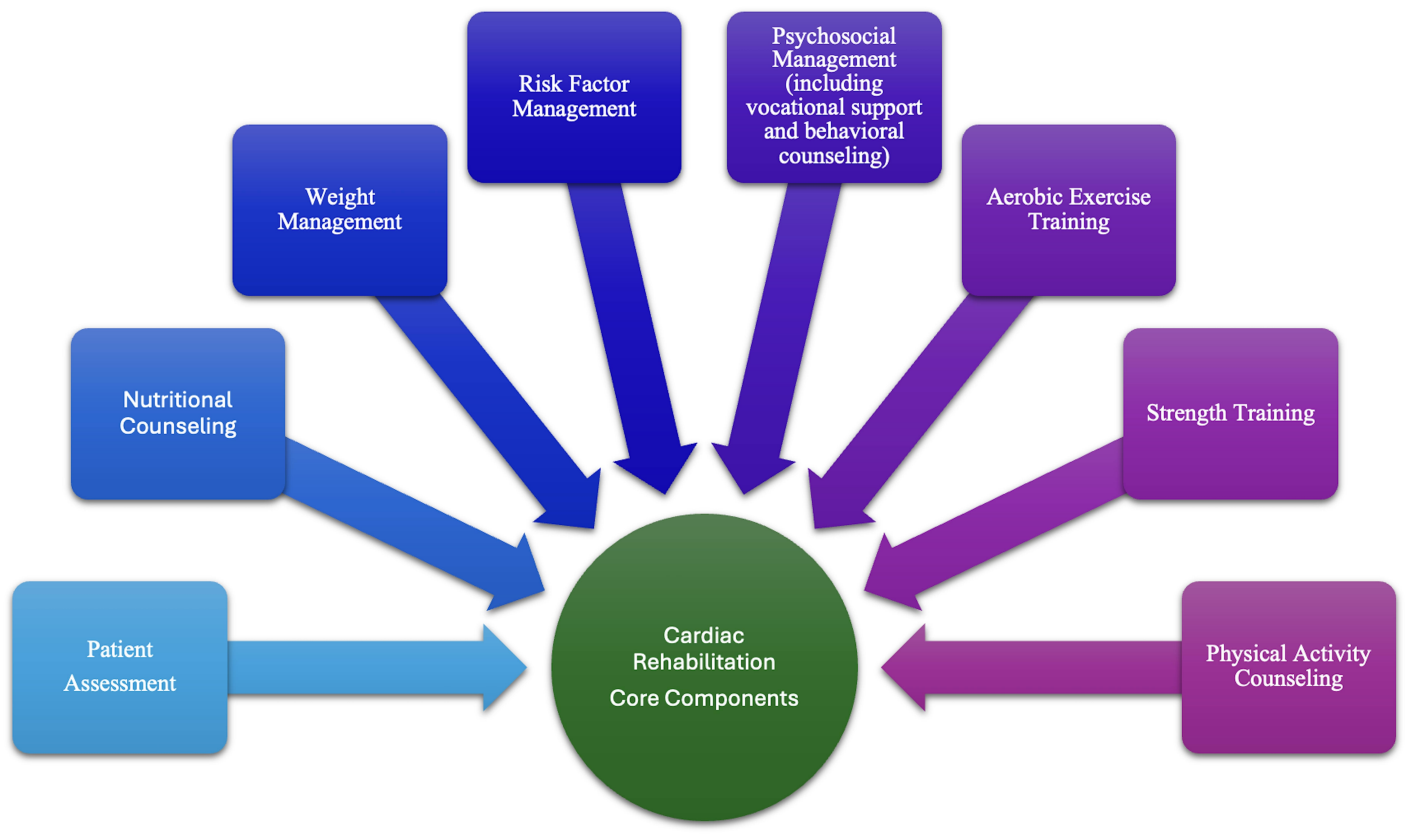
**CR components**. The components needed for a successful CR program are shown. CR, cardiac rehabilitation.

A typical core structure of CR includes the following components: initial patient assessment, supervised aerobic and resistance exercise training, dietary counseling, risk factor modification (e.g., blood pressure, lipids, glycemic control, smoking cessation), and psychosocial support [3]. Psychosocial management includes vocational support and behavioral counseling. Specifically, the American Heart Association recommends that CR programs assess psychosocial management through validated screening tools, intervene through individual or group counseling, and create a supportive CR environment [11]. While the standard model in the United States comprises 36 sessions over 12 weeks, regimen intensity and composition vary based on patient functional status and comorbidities [9]. The integration of both aerobic and resistance training has been shown to yield superior improvements in muscular strength and exercise prescription should be stratified according to baseline functional status and cardiac risk [3, 13]. Risk factor modification is another cornerstone of CR, and interventions can be focused on targeting hypertension, hyperlipidemia, diabetes, obesity, and tobacco use. Furthermore, the modalities of CR can be delivered through traditional center-based programs, home-based models, or hybrid approaches employing telemonitoring. Traditional center-based models typically offer real-time ECG monitoring and exercise supervision. Fig. 1 shows the many components needed for a successful CR program [11].

CR is known to be safe, with one study assessing cardiovascular events in 30 CR programs showing that there was one fatal event every 116,402 hours of patient participation and one non-fatal event every 34,673 hours of patient participation [14]. However, patients have been shown to develop musculoskeletal comorbidities after starting CR, with one study showing that 15% of patients at 3 months developed musculoskeletal issues with a large portion of them being sprains [15].

#### 2.1.1 Exclusion Criteria for CR

The presence of active uncontrolled cardiovascular disease is a common contraindication of CR. This is based on physiologic risk during exertion and the need for clinical stability. Common exclusions include active unstable angina, decompensated heart failure, severe aortic stenosis, uncontrolled arrhythmias, recent thromboembolic events, and significant cognitive impairment [3, 9]. Severe aortic stenosis poses a high risk for exertional syncope and sudden cardiac death due to fixed cardiac output limitations. Uncontrolled arrhythmias, particularly ventricular tachyarrhythmias, increase the risk of sudden cardiac events during physical activity. Cognitive or psychiatric impairments function as contraindications as safe participation must be assessed. Most exclusions are temporary, and re-evaluation for CR is commonly recommended once patients achieve medical stability.

#### 2.1.2 Standardized Metrics in CR

To assess functional improvement and program efficacy after initiation, individualized CR is assessed through a range of standardized metrics. Exercise capacity is commonly quantified using metabolic equivalents (METs) obtained from treadmill or cycle ergometer testing, with increases of 1–2 METs correlating with substantial reductions in cardiovascular mortality [16]. Dartmouth COOP (cooperative functional assessment charts) assess patient-reported functional status across domains such as physical fitness, emotional well-being, pain, and daily activities, offering a validated, low-burden quality-of-life instrument [17]. The Patient Health Questionnaire-9 (PHQ-9) is routinely used to screen and monitor depressive symptoms, which are prevalent in CR populations and associated with adverse cardiovascular outcomes [18]. Additional tools include the 6-minute walk test to measure submaximal functional capacity, blood pressure, lipid panels, and behavioral metrics such as smoking status and medication adherence scales [3]. Despite broad consistency, some trials report attenuated or non-significant clinical effects which may largely be attributable to methodological limitations. For instance, in HF-ACTION, the primary endpoint (all-cause mortality or hospitalization) did not achieve statistical significance, possibly due to low adherence [19].

### 2.2 CR Outcomes

#### 2.2.1 Coronary Artery Disease and Revascularization 

Accumulating observational data and clinical trials from the 1970s through the 1990s showed that CR participation significantly reduced all-cause and cardiovascular mortality in post-MI patients [1, 2]. Rehabilitation following acute coronary syndrome has significantly limited cardiovascular morbidity and mortality [1, 2, 3]. Current evidence supporting CR in stable angina remains limited due to a paucity of large-scale trials [20]. A meta-analysis of 63 studies by Anderson *et al*. [21] demonstrated a relative risk reduction in cardiovascular mortality of 0.74 and in hospital admissions a relative risk reduction of 0.82 with exercise-based CR compared to usual care for those with coronary heart disease. Similarly, a retrospective cohort study by Suaya *et al*. [22] reported a 21% reduction in 5-year mortality among Medicare beneficiaries who initiated CR. Beyond mortality and readmissions, CR also improves psychosocial outcomes in predominantly acute coronary syndrome and revascularized patients: a registry analysis of 27,670 patients by Quindry *et al*. [23] found that mean PHQ-9 score reductions of 40–48% post-CR. One study assessing CR timing and duration after PCI reported no significant improvements in arrhythmia rates, restenosis, angina, left ventricular ejection fraction, or 6-minute walk distance, suggesting that the timing of CR initiation alone may be insufficient to affect these outcomes without consideration of program intensity, patient adherence, and baseline cardiovascular risk [24]. As a result, it is clear that CR offers significant mortality and psychosocial benefits, but the timing and duration of CR may not have a large role in those improvements.

#### 2.2.2 PAD

CR has been a validated secondary prevention strategy in symptomatic PAD, with multiple trials demonstrating functional gains. Gardner *et al*. [25] demonstrated that patients with PAD who participated in a supervised exercise program showed a significant increase in claudication onset time and in peak walking time compared to controls. Similarly, McDermott *et al*. [26] demonstrated in the GOALS trial that a home-based walking and cognitive behavioral program significantly improved various exercise metrics, including improvements in the 6-minute walk distance with a mean increase of 53.5 meters in PAD patients. Moreover, the CLEVER trial compared supervised exercise, stenting, and optimal medical therapy, and it showed that supervised exercise yielded the largest improvement in treadmill walking time of 5.8 minutes compared to 3.7 minutes with stenting and 1.2 minutes for optimal medical therapy [27]. As a result, structured walking-based CR programs have been shown to attenuate the progression of early claudication symptoms while significantly enhancing ambulatory function and overall symptom burden.

#### 2.2.3 Heart Failure

CR is increasingly becoming recognized as a key strategy for secondary prevention for patients with HFrEF. In the HF-ACTION trial, exercise-based CR led to a modest but significant improvement in peak oxygen uptake and in the 6-minute walk test of 20 meters compared to 5 meters [19]. Taylor *et al*. [28] showed that exercise-based CR in patients with HFrEF resulted in a significant improvement in health-related quality of life across 13 trials (1270 patients), as evidenced by a mean reduction of 5.8 points in the Minnesota Living with Heart Failure questionnaire. The REHAB-HF trial further showed that early in-hospital initiation of individualized physical rehabilitation led to a 1.5-point improvement in the Short Physical Performance Battery, showing that CR can improve physical function in older adults with acute decompensated heart failure [29]. As a result, these trials show that CR improves exercise and physical function metrics in HFrEF patients, but based on the current evidence, it is unclear if these benefits translate to improvements in mortality [19, 29].

#### 2.2.4 Cardiac Valvular Repair

More recently, CR has been increasingly recognized as a useful adjunct in enhanced recovery and reduced readmissions for patients after valvular repair. Butter *et al*. [30] showed that in 1056 elderly patients who underwent transcatheter aortic valve implantation, participation in a 3-week CR was associated with a significant reduction in mortality compared to no intervention. RECOVER-TAVI is a randomized trial that investigated the functional metric outcomes of CR in transcatheter aortic valve implantation patients receiving CR with no apparent differences between groups at the 3 and 6-month follow-up period [31]. There is limited literature regarding the impact of CR on patients who underwent cardiac valvular repair, and further studies are needed to understand CR’s impact on patient outcomes in this population.

#### 2.2.5 Heart Transplantation

Cardiac transplant recipients exhibit abnormal exercise physiology due to surgical cardiac denervation, persistent diastolic dysfunction, and residual impairments from pre-transplant chronic heart failure, including diminished skeletal muscle oxidative capacity and attenuated peripheral vasodilatory response [32]. A retrospective cohort study by Bachmann *et al*. [33] demonstrated a 29% reduction in 1-year all-cause readmission among Medicare beneficiaries who participated in CR after heart transplantation. Despite recommendations from professional societies endorsing supervised CR pre- and post-transplant, high-quality evidence supporting functional and survival benefits remains sparse. Greater variation in the modality of CR programs and individual tailoring to patient needs may be more difficult to study in complicated cardiovascular conditions, such as transplantations.

#### 2.2.6 Preventive CR Outcomes

Preventive CR has also been shown to improve outcomes in high-risk individuals [34, 35]. Patients with type two diabetes mellitus and coronary artery disease have been shown to have improved exercise capacity, endothelial function, and waist circumference with exercise interventions [34]. Similarly, another study found that type two diabetes mellitus patients who underwent CR also had improvements in their exercise capacity, glycemic control, and blood pressure [34, 35]. CR has also been shown to have an impact on weight [36]. One study of obese and overweight patients who underwent CR showed that 27% of subjects reduced their weight by 3% or more [36]. In another study assessing obese patients who enrolled in CR, mean weight loss was modest with 0.9 kg loss in men and 0.5 kg loss in women [37]. Increased initial weight and age were predictors of weight loss [37]. Dun *et al*. [38] showed how high-intensity interval training in CR patients after MI had decreased metabolic syndrome components, body fat mass and increased lean mass. This study shows that high-intensity interval training may have unique metabolic and compositional benefits within CR [38].

### 2.3 CR Underutilization and Disparities

#### 2.3.1 Reasons for Underutilization and Disparities

Although CR referral rates have been increasing over time [39], overall rates of CR utilization are low [6, 40]. One study showed only 16.3% of qualifying Medicare patients and 10.3% of Veterans Affairs patients participated in CR [6]. A different study showed that only 28.6% of patients underwent a CR session [40]. As a result, the majority of patients do not receive the many benefits of CR. Furthermore, there is a large variation between US states ranging from 3.2% and 41.8% participation rates between states in Medicare patients with those in the Pacific region generally having the least participation [6].

One possible reason for the variations is the presence of CR deserts, which are determined through hotspot analysis, which can make it difficult for patients living in those deserts to find a place to undergo rehabilitation [41]. As expected, being closer to a CR facility is a predictor of CR utilization [42]. Given that the patients who qualify for CR usually have major comorbidity or have recently undergone a major operation, many of them may depend on others for transportation or may not be able to travel large distances. As a result, they may be unable to attend the CR sessions.

Increased rates of CR utilization were seen in those with a higher household income and education level [42]. Patients living in deprived communities, as measured by a neighborhood deprivation index, were less likely to start CR [43]. Another study similarly showed that CR referral predictors were white race and hospital bed size [39]. Non-white, elderly, and female patients have been shown to have decreased rates of CR usage [42]. Only 18.9% of women participated in CR compared to 28.6% of men [44]. Furthermore, only 13.2% of Hispanic people and 13.6% of black people participated in CR compared to 25.8% of non-Hispanic white people [44]. Provider-level barriers including under-referral for minorities and women, and the strength of CR endorsement by physicians also contribute to underutilization [12]. As a result, it is clear that vulnerable populations are underutilizing CR. This may be due to systemic issues and patients not receiving the appropriate education about the importance of CR within these groups. This may also be due to these groups, especially those from a lower socioeconomic status, being unable to take time off to attend CR sessions. Furthermore, dropout rates of CR are high [44, 45]. One study reported a 27% completion rate, but other studies report varied rates based on different methodologies [44, 45]. Reasons that contribute to the high dropout rates include caregiver responsibilities, lack of education regarding CR benefits, financial barriers, and transportation [44, 45].

Between hospitals, there was also a large variation in Medicare patients, ranging between 3% and 75% [6]. Given the wide range of usage between hospitals [6], it is likely that there are multiple reasons for the underutilization. There might be a lack of education among discharging providers regarding the importance of CR. Furthermore, the burden may be on patients themselves to find CR sites without any assistance from the hospital itself. Female patients have been shown to receive 12% fewer CR referrals [46]. This could be due to providers not realizing the impact that CR can have on women. Furthermore, female patients have been shown to have lower rates of CR participation of 18.9% compared to 28.6% in men [44]. As a result, even the women who are referred are participating in CR at a lower rate.

#### 2.3.2 Interventions to Address Disparities and Underutilization

Interventions that have been shown to increase CR utilization include appointments close to the discharge date, regimens that are gender-specific, and motivational letters [47]. Setting up CR appointments close to the discharge date may improve CR utilization because the preceding event, whether it be a recent PCI or a new diagnosis of major cardiac morbidity, is likely on the patients’ mind and they may be motivated to prevent the recurrence of those issues or worsening of their condition. Gender-specific regimens may also improve CR utilization because patients may feel like the exercise is more tailored to them. Motivational interviewing has been shown to be associated with a 25% higher CR completion rate [48]. Motivational interviewing for CR may help patients remind themselves of why they are doing these exercises. CR adherence improved with individual counseling by staff and individuals recording their activity [47]. One study showed that among patients who received learning and coping strategies, which involved the use of patients who previously underwent CR as educators and individual interviews, there was increased adherence to CR [49]. There were increased effects of these strategies on adherence in patients with a lower household income and education [49].

**Fig. 2.**
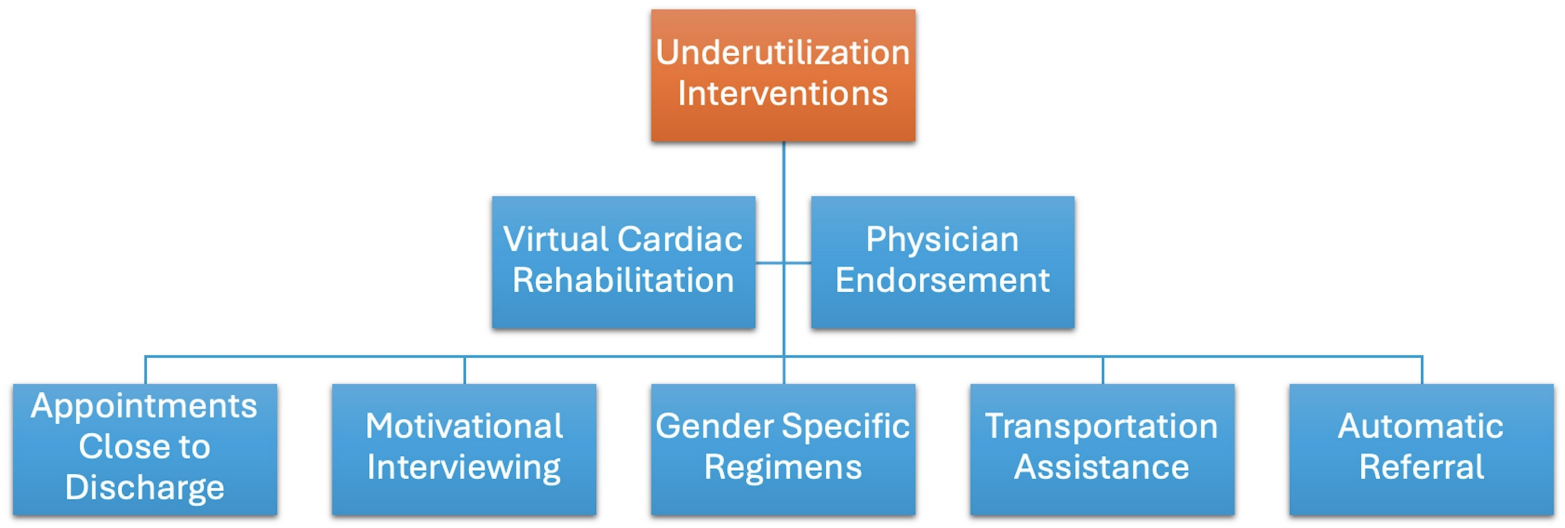
**Interventions to address CR underutilization**. Interventions that can address the underutilization of CR are shown.

A stronger endorsement of CR by physicians was also shown to affect CR utilization [50]. As a result, it is clear that while there are many societal factors involved in patients’ CR usage, there is still room for improvement in provider education about CR. Furthermore, given the lower CR referral rate of female patients [46], further reinforcement of providers encouraging everyone who may benefit from CR to go could increase CR enrollment. Combining both an automatic CR referral on discharge and having a CR referral discussed with a healthcare professional or peer graduate also increased CR utilization [51, 52]. Another potential solution to CR underutilization is providing transportation to CR or travel reimbursements [53]. Automatic referrals after a qualifying event and assisting with transportation are all ways to decrease friction in the CR enrollment process, and can hopefully increase CR completion rates. Fig. 2 shows interventions that can address the underutilization of CR.

### 2.4 VCR Methodology 

VCR is an expanding field and is a possible remedy to the underutilization of CR. Some studies show similar outcomes of VCR to traditional CR [7, 8]. We detail below the methodology of the recent major VCR studies. We report this methodology given that VCR regimens often vary between institutions. Shah *et al*. [54] is one of the largest recent studies looking at 703 VCR patients and comparing them to 2303 traditional CR patients. VCR patients were sent a tablet, blood pressure monitor, exercise bands, and a heart rate monitor [54]. They also engaged in 36 video sessions that happened 2 to 3 times a week across 12 and 18 weeks [54]. These sessions included group exercise sessions, well-being discussions, and nutrition counseling [54]. These sessions were supervised by a cardiologist with exercise physiologists and dieticians having a more direct role [54]. Some VCR exercises included leg raises, stepping in place, resistance band exercises, and standing up from sitting exercises [54].

Hilu *et al*. [8] is another major recent study that included 107 VCR and 198 traditional CR patients [8]. VCR patients were sent smart watches for physical activity tracking. They communicated through a mobile phone app, and their activity was monitored by staff [8]. Every 3 months, patients were assessed by a cardiologist through a clinical assessment and measurement of body fat percentage and treadmill exercise test performance [8]. Ganeshan *et al*. [55] also involved 37 traditional, 38 hybrid, and 20 VCR participants who completed the program. All three groups received training and counseling from the same providers, cardiologists, and physiologists [55]. VCR sessions were conducted through video meetings (Zoom) and were 31–60 minutes for 10 sessions [55]. For those who could not utilize this video conference, a telephone was used [55]. Mobile phone applications also allow for tracking and sending of reminders and educational materials [55]. Hybrid patients had approximately 9 in-person sessions and 7 video or phone sessions [55].

Some of the larger modern VCR studies’ methodology was discussed above. There were other VCR studies published over the past few years [56, 57, 58, 59, 60, 61]. Some commonalities between VCR regimens at programs include an initial in-person session to determine individualized training plans and assess clinical metrics like weight and BMI. Similarly, most recent VCR studies involve some type of video conferencing as well as a virtual mobile application in which exercise can be logged. Supervision with exercise physiologists and the availability of a provider, if needed, is another commonality between many of the VCR programs. However, there are also many notable variations between the programs. They range in the timespan of each session and the total number of sessions. They also vary in exercises, with some using treadmills and other exercise equipment that patients already have at home and others involving exercises that do not require equipment. Some studies also incorporated ancillary staff such as dieticians and motivational interviewing while some did not. As a result, there is a large variation among VCR programs, and this is important to consider when reading reviews and meta-analyses generalizing VCR findings.

### 2.5 VCR Outcomes

#### 2.5.1 Exercise and Body Metrics

Some studies show similar outcomes of VCR to traditional CR [7, 8]. Similar 6-minute walk test and blood pressure control improvements have been seen in traditional CR when compared to VCR and hybrid CR modalities [55]. More patients had at least a 10% increase in exercise capacity in VCR patients (69.3%) compared to traditional CR (33.8%) [8]. Other VCR studies have been published over the past few years as well [56, 57, 58]. Although there is variability in how exercise metrics change before and after VCR compared to traditional CR, most studies show that VCR is not inferior or possibly even superior to traditional CR for certain metrics. Similarly, other metrics including patient BMI and blood pressure have been compared. VCR has comparable changes to traditional CR in systolic and diastolic blood pressure [62]. VCR had a greater reduction in body fat percentage change compared to traditional CR [8]. Traditional and VCR patients had no difference in change in BMI or muscle mass percentage change [8]. Once again, most studies show similar findings regarding changes in body composition and vitals between traditional CR and VCR.

#### 2.5.2 Patient Outcomes and Quality of Life Metrics

Patient clinical outcomes after VCR have also been measured, although there is limited research on this topic given the nascency of the field. VCR has been shown to have reduced hospital readmission and emergency department (ED) admission rates [54]. There was no difference in mortality or MI rates [54]. Once again, VCR does not appear to be inferior to traditional CR regarding clinical outcomes although more research is needed before guidelines can categorize VCR as non-inferior to traditional CR. Hybrid CR has been shown to have a higher change in Dartmouth COOP scores, showing that hybrid CR may even report greater improvements in quality of life compared to traditional CR [63]. One study reported VCR patients had a smaller depression symptom improvement compared to traditional CR [55]. A different study showed that PHQ-9 scores improved in both traditional and hybrid VCR [64]. VCR and hybrid CR patients were found to have similar improvement in Generalized Anxiety Disorder-7 scores [55]. As a result, the data regarding quality-of-life metrics comparing VCR and traditional CR is more varied. There is also limited literature regarding these topics. Further research will be needed examining these metrics to determine VCR’s impact on quality of life.

#### 2.5.3 Feasibility and Patient Preferences

In one study, 91.0% of patients found that it was not difficult to coordinate exercises with VCR instructions provided through their devices [56]. Furthermore, 80.9% of patients felt safer engaging in VCR compared to when they did exercise without supervision [56]. As a result, it is clear many patients are able to undergo VCR and feel comfortable engaging in exercise in this modality [56]. However, having virtual sessions has its drawbacks as well. In one study, 62.8% of participants reported that the sound quality was not satisfactory [56]. Furthermore, 39.3% of participants were unable to attend a VCR session because of technical issues [56]. An American Heart Association advisory highlights that increasing training on how to use digital technology will be important in preventing exploitation of patients with low digital literacy as VCR becomes more common [59]. This is because without this training, patients with low digital literacy may, on the surface, be receiving VCR, but their experience may be plagued by technical issues even though they are paying the social and financial costs of attending VCR [59]. This American Heart Association advisory discusses other challenges of VCR, including reimbursement uncertainty as well as how it will be important to develop digital dashboards to increase patient safety [59]. One large-scale VCR study demonstrated high VCR program adherence throughout the study duration [54]. However, more research is needed to understand VCR adherence rates [54]. Another challenge of digital modalities like VCR is the absence of clear regulations regarding the implementation of this technology and variations in state regulations that make widespread adoption more difficult [65].

**Fig. 3.**
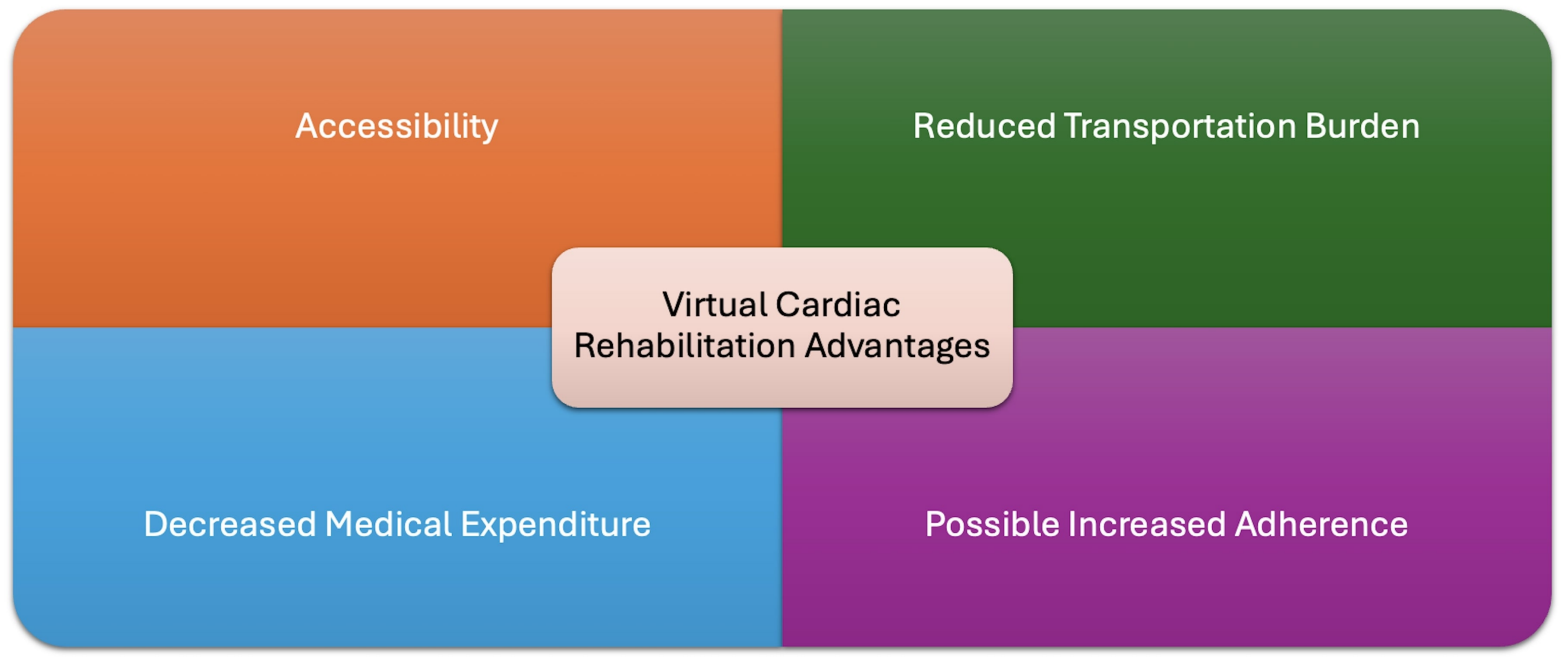
**VCR advantages**. This figure shows the primary advantages of VCR over CR.

As a result, VCR is not for everyone, and those who have limited experience with technology or those who have limited internet access may not be good candidates for VCR [56]. Lower medical expenditure is associated with VCR [54]. It is clear to see where the cost savings may result through the increased utilization of VCR as there is no need to maintain in-person rehabilitation centers and in-person exercise equipment. Fig. 3 shows the primary advantages of VCR.

When given a choice between traditional and VCR, patients reported choosing equivalent proportions of each [66]. Patients have also been shown to complete more sessions through VCR with one study showing VCR patients underwent 34.4 sessions compared to 21.4 sessions through traditional CR from a total of 36 sessions [54]. As a result, the decision to pursue VCR or traditional CR is highly personalized and may impact how adherent they are to completing the CR. We recommend that patients have the option to choose given the possible advantages of VCR including increased access, easier scheduling, decreased transportation, more privacy, and decreased delays in enrollment [3]. These factors may allow some patients to feel more comfortable with VCR. However, some possible disadvantages of VCR include decreased social support, gaps in reimbursement, safety concerns, less well-researched outcomes, and decreased patient accountability [3]. These disadvantages may be why some patients may prefer traditional CR. Given the number of variables involved in deciding whether VCR or traditional CR is more suitable for patients, we recommend that providers explain these advantages and disadvantages and also explain outcomes seen with the two modalities to allow the patients to choose the regimen that meets their needs best.

### 2.6 Current CR Research

There are several ongoing CR trials. The mTECH-Rehab will include 200 patients randomized to receive usual care or enrolled in a program involving a mobile application focused on patient empowerment, smart devices to collect metrics, and virtual coaching sessions [67]. This trial aims to add to the growing trend in the literature focused on CR utilizing smart devices and integrating home experiences [67]. Another ongoing trial is the SWEDEHEART study which is a randomized crossover clinical trial that plans to include 1500 participants [68]. This trial aims to determine if VCR is as effective as traditional CR and determine if it increases participation [68]. Monitoring of major adverse cardiovascular events at 1 and 3 years will also be assessed to determine the clinical impact of VCR compared to traditional CR [68]. Other CR trials are also ongoing and focus on various solutions for underutilization [69, 70, 71, 72]. The PRO-FIT trial takes a different approach and is trying to answer whether CR is as effective as usual care (such as coronary vascularization) at decreasing anginal pain in patients with stable angina pectoris [73]. As a result, over the next few years, we expect there to be clearer outcomes regarding long-term clinical outcomes of CR for various indications and more solutions to CR underutilization.

### 2.7 Areas for Future Research

Although there is an increasing amount of research being published, there are many gaps in knowledge in the current CR literature. There are many indications for CR referral, and the indications for CR referral have grown over time [3]. However, there is less research about other indications for CR such as atrial fibrillation or for those with congenital heart disease. CR may decrease atrial fibrillation symptom severity and burden [74]. Another study showed that CR improved exercise capacity in those with congenital heart disease [75]. Conducting further research to determine new indications for CR might allow patients to experience gains in exercise metrics and improved clinical outcomes. There has also been research over many decades about the long-term clinical outcomes of CR. However, there is less research regarding quality-of-life metrics such as changes in Dartmouth COOP scores or depression and anxiety scores. Furthermore, there is limited research regarding change in quality-of-life outcomes stratified by each indication. Research into these quality-of-life metrics could allow providers to understand the impact of CR outcomes and, in turn, could increase patient compliance with CR if they understand how their lives might change because of CR.

CR is underutilized, and reasons for the underutilization have been described. However, there remains limited literature about solutions to address many reasons for the underutilization. Some solutions such as automatic referrals and motivational interviewing have been described, but research regarding the implementation of other strategies targeting underutilization like transportation reimbursement programs is limited. Further research into underutilization solutions of CR could improve patient outcomes as more patients would be able to complete the CR regimens. VCR research is an expanding field with CR, but there remain few large-scale studies comparing VCR to traditional CR. The data regarding quality-of-life metrics comparing VCR and traditional CR is more varied, and there is limited literature regarding these topics. More research into safety concerns regarding VCR and establishing clinical outcomes could allow for the wider adoption of VCR.

## 3. Conclusions

The advantages of CR have been established as beneficial for secondary prevention after major cardiac interventions or in those with major cardiac morbidities [4, 5]. Despite the many benefits of CR and although CR referral rates have been increasing over time [39], overall rates of CR utilization remain low [6, 40]. It is also clear that vulnerable populations are underutilizing CR [39, 42, 43]. Interventions that have been shown to increase CR utilization include appointments close to the discharge date, regimens that are gender-specific, and motivational letters [47]. Automatic referrals after a qualifying event and assisting with transportation are all ways to decrease friction in the CR enrollment process.

VCR is an expanding field and is a possible remedy to the underutilization of CR. There is a large variation among VCR programs, and this is important to consider when reading reviews and meta-analyses generalizing VCR findings. This is also important for providers who are deciding whether a patient might be a good fit for VCR to understand what specific regimen their patients would be enrolling in. Although there is variability in how exercise metrics change before and after VCR compared to traditional CR, most studies show that VCR is not inferior.

However, VCR is not for everyone, and those who have limited experience with technology or those who have limited internet access may not be good candidates for VCR [56]. Widespread VCR implementation is further limited by variations in reimbursement and state laws regarding VCR [59, 65]. Given the number of variables involved in deciding whether VCR or traditional CR is more suitable for patients, we recommend that providers explain these advantages and disadvantages. Given the clear beneficial outcomes of CR, we encourage all provider to have a vested interest in ensuring their patients can access CR. Furthermore, we encourage more research to address CR underutilization so more patients can experience the improved outcomes seen with CR.
